# The contemporary role of renal mass biopsy in the management of small renal tumors

**DOI:** 10.3389/fonc.2012.00106

**Published:** 2012-09-10

**Authors:** Amy Lim, Brock O'Neil, Marta E. Heilbrun, Christopher Dechet, William T. Lowrance

**Affiliations:** ^1^MD/PhD Program, University of UtahSalt Lake City, UT, USA; ^2^Division of Urology, Department of Surgery, Huntsman Cancer Institute, University of UtahSalt Lake City, UT, USA; ^3^Department of Radiology, University of UtahSalt Lake City, UT, USA

**Keywords:** renal mass, renal neoplasms, renal cell carcinoma, renal biopsy, nephrectomy

## Abstract

The selective use of percutaneous biopsy for diagnosis in renal masses is a relatively uncommon approach when compared to the management of other solid neoplasms. With recent advancements in imaging techniques and their widespread use, the incidental discovery of asymptomatic, small renal masses (SRM) is on the rise and a substantial percentage of these SRM are benign. Recent advances in diagnostics have significantly improved accuracy rates of renal mass biopsy (RMB), making it a potentially powerful tool in the management of SRM. In this review, we will discuss the current management of SRM, problems with the traditional view of RMB, improvements in the diagnostic power of RMB, cost-effectiveness of RMB, and risks associated with RMB. RMB may offer important information enabling treating clinicians to better risk-stratify patients and ultimately provide a more personalized treatment approach for SRM.

## Introduction

**Figure 1:**
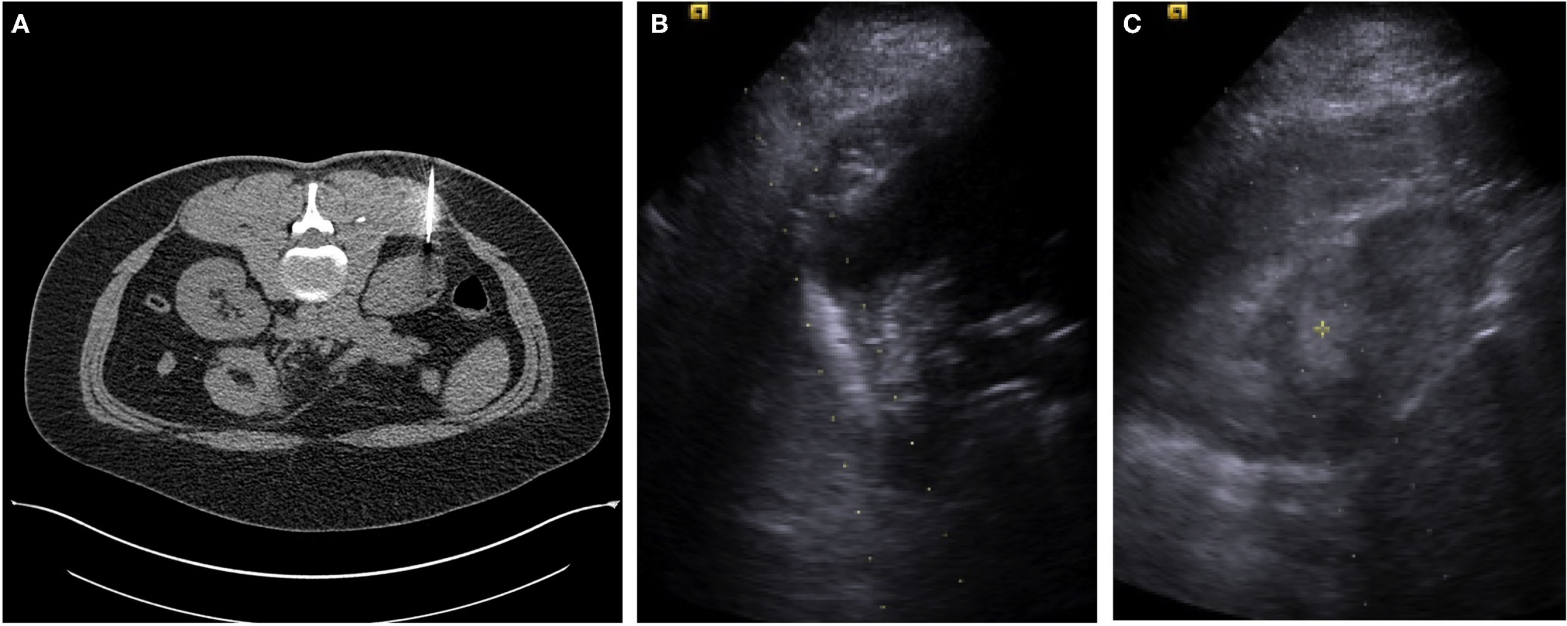
**(A)** A single axial CT image from a percutaneous CT guided biopsy using a coaxial technique and a 22-guage needle for fine needle aspirate sampling in a female with an incidentally detected 2.7 cm lower pole R renal mass. Pathology reported renal cell carcinoma, without subtyping. **(B)** An ultrasound image with the needle guide in place of a male with an incidentally detected 3.5 cm right upper pole renal mass. **(C)** A second ultrasound image from a percutaneous US guided biopsy using a coaxial technique and an 18-guage needle for core biopsy. Pathology reported as renal cell carcinoma, conventional clear cell type, Furman grade I–II.

In 1971, a review of 309 cases reported that 60% of patients with renal cell carcinoma presented with hematuria, 30% with flank pain, and 25% with an abdominal mass. The classic triad of all three was seen in 9% of patients while only 7% were asymptomatic (Skinner et al., 1971). Technological advances have resulted in new methods for detecting and treating renal masses. Now most are discovered incidentally on CT scan (Figure 1) with only 24% of patients with hematuria, 10% with flank pain, and 8% with an abdominal mass (Jayson and Sanders, 1998). The classic triad is now rarely seen (0.7%) and 61% are asymptomatic at detection (Jayson and Sanders, 1998). At the same time the incidence of primary renal malignancies has been steadily increasing in the United States over the past two decades (Chow et al., 1999), and the average size of renal masses discovered at presentation is getting smaller (Nguyen et al., 2006).

Small renal masses (SRM) are defined by a greatest diameter of 4 cm or less and constitute 48–66% of all newly diagnosed renal tumors and 38% of all excised renal masses (Lee et al., 2000; Nguyen et al., 2006). The rising trend in the diagnosis of primary renal malignancies and the detection of renal masses at smaller sizes is due in part to increasing utilization of sophisticated diagnostic imaging modalities.

While the logical result of this trend toward an apparent early detection of more renal masses at a smaller size would be improved cancer specific survival rates for primary renal malignancies, this has not been realized with slightly increasing renal cell carcinoma mortality rates (Hollingsworth et al., 2006). This suggests that our current treatment paradigm towards renal tumors or renal masses may not be the most effective for preventing death from kidney cancer. Additionally, pathologic staging shows that approximately 20–50% of SRM are benign, and a subgroup of the malignant masses are likely indolent (Frank et al., 2003; Nguyen et al., 2006; Russo, 2008). Therefore, the benefits of aggressive surgical management for some SRM may not always outweigh the associated risks.

One long discussed, but infrequently utilized approach to help selectively apply the benefits of surgical management without over treatment is the renal mass biopsy (RMB). In this review, we will discuss the current management of SRM, problems with the traditional view of RMB, improvements in the diagnostic power of RMB, cost-effectiveness of RMB, and risks associated with RMB, arguing that RMB offers important information for treating clinicians to risk-stratify patients with SRM.

## Treatment options for SRM

### Surgical management: radical and partial nephrectomy

Localized solid SRM are typically treated with either nephron-sparing surgery (NSS) or radical nephrectomy. Other management options include active surveillance (AS) or ablative (radiofrequency or cryoablation) therapies. For renal lesions that are amenable to NSS, partial nephrectomy is typically favored over radical nephrectomy for several reasons. First, rates of chronic kidney disease (CKD) are rising and partial nephrectomy clearly preserves renal function compared to radical nephrectomy (Coresh et al., 2007). This benefit was demonstrated by Huang et al. who retrospectively analyzed 662 patients with normal baseline renal function and subsequently underwent partial or radical nephrectomy for SRM. Only 3% of these patients who underwent partial nephrectomy developed new onset of CKD compared to 36% of patients who underwent radical nephrectomy. Second, NSS results in similar oncological control when compared to radical nephrectomy (Becker et al., 2006; Thompson et al., 2008). Third, radical nephrectomy and partial nephrectomy have similar complication rates (Stephenson et al., 2004; Lowrance et al., 2010).

Although partial nephrectomy is the preferred surgical treatment for SRM, according to Surveillance, Epidemiology and End Results data, only 35% of SRM are removed by this method while the remaining 65% continue to undergo radical nephrectomy (Dulabon et al., 2010). Underutilization of partial nephrectomy is likely multifactorial, but may be partly due to the comfort level of surgeons performing this technically demanding procedure or to patient access to tertiary care centers providing this service. Underutilization of nephron-sparing treatments is concerning, given that approximately 20–50% of renal masses are benign, placing a sizeable group of patients at risk for CKD for a procedure that may be unnecessary (Russo et al., 2012). RMB may help elucidate which patients are likely to benefit from extirpative therapy and reassure clinicians monitoring patients who are not.

### Non-surgical options: active surveillance and ablation

AS and image-guided tumor ablation are non-traditional treatment options for SRM. Typically, these modalities are reserved for patients with complicated coexisting morbidities, limited life expectancy, have other significant surgical risk factors, or due to patient preference. However, AS may be suited for additional groups if clinicians were better able to risk stratify patients.

AS is especially appealing for smaller masses as it has been shown that small size (Frank et al., 2003) and slow growth rates (Kouba et al., 2007; Abouassaly et al., 2008; Boorjian and Uzzo, 2009; Jewett et al., 2011; Smaldone et al., 2012) correlate with a low malignancy potential. Further, evidence suggests that malignancy rates for patients managed by AS are not statistically different from select patients managed through partial nephrectomy or ablative therapies (Abou Youssif et al., 2007; Kunkle et al., 2007), and delayed surgical intervention as a result of AS does not appear to compromise surgical outcomes (Kouba et al., 2007; Boorjian and Uzzo, 2009). Collectively, these data suggest that AS is an option for SRM in select patients, although randomized studies comparing AS to early intervention for SRM with long-term follow-up are needed to fully endorse this approach.

Image-guided tumor ablation is an additional treatment option with favorable short-term outcomes. However, long-term robust oncological data is not yet available. Berger et al. (2009) reported reasonable 5 and 10-year radiographic cancer-specific survival rates at 93 and 81%, respectively. Yet these rates are less successful compared to partial nephrectomy, where five and 10-year cancer-specific survival rates are as high as 96 and 90%, respectively (Chawla et al., 2006).

Radiofrequency ablation is also utilized for primary management of SRM but is reported to have higher recurrence rates when compared to cryoablation. This difference does not appear to affect metastatic progression (Kunkle and Uzzo, 2008; Heuer et al., 2010). One must also consider that prior ablative therapy may complicate salvage therapy by partial nephrectomy due to significant fibrosis (Crowley et al., 2001; Zhu et al., 2006; Nguyen and Campbell, 2008; Kowalczyk et al., 2009). Although surgical excision has better long-term oncologic outcomes, RMB may be helpful in guiding treatment decisions for patients considering other options.

## Problems with the traditional view of renal mass biopsy

RMB is currently uncommonly used in the evaluation of newly discovered SRM. In one population-based medical claims analysis, only 6% of patients undergoing nephrectomy (partial or radical) for a renal tumor had a preoperative renal biopsy performed within the 6 months prior to surgery (Lowrance et al., 2011). Conventional thinking about the value of percutaneous renal biopsy has deterred most physicians from utilizing this as a diagnostic tool in the decision of how to manage SRM. This is at least partly due to reported false positive rates of up to 5%, but more significantly, false negative rates as high as 25%. Physicians may also be hesitant to biopsy if their institution lacks Interventional Radiologists that routinely perform this procedure, which would inherently affect the rate of failed biopsies. With reported failure rates of 0–22% prior to 2001 and 0–18% from 2001 onward (Lane et al., 2008), it is reasonable that physicians would take a more conservative approach in utilizing biopsy as a diagnostic procedure. Finally, there is a strong belief that RMB will not change management (Khan et al., 2007).

High false negative rates are slightly misleading and outdated as underscored by a meta-analysis of 2474 renal mass biopsies by Lane et al. First, the false-negative rates were originally calculated from both failed biopsies and misinterpreted results as opposed to only negative results. When these biopsies were reanalyzed and more appropriately categorized as technical failures instead of negative test results, the false-negative rates and positive rates were 4.4 and 1.2%, respectively. Second, if the first biopsy is not helpful, repeat biopsy can be utilized to decrease the rate of biopsy failures for indeterminate or failed biopsies (Murphy et al., 1985; Nadel et al., 1986; Wood et al., 1999; Shannon et al., 2008). Third, false positive and negative rates are improving with advancements in imaging, biopsy technique, immunohistochemistry and new molecular markers (see section “Advancements in RMB” below).

With improved false-negatives rates and current reported sensitivities of 80–92% and specificities of 83–100% (Rybicki et al., 2003; Volpe et al., 2007; Shannon et al., 2008), it is hard to believe that RMB would not change management. Nonetheless, historic studies report a wide range of change in management (6.3–47.8%) based upon response to core biopsy results. Moreover, inconsistent definitions of “change in management” are so varied that it is often difficult to make meaningful conclusions from studies that evaluate this criteria (Rybikowski et al., 2008; Shannon et al., 2008; Thuillier et al., 2008). Yet, others have reported that RMB provided important information in determining which patients would be best for AS, NSS, or radical nephrectomy based upon indeterminate, benign, intermediate, or unfavorable histology (Tan et al., 2012). Unfortunately they do not report the favored management choice prior to RMB, limiting conclusions from this type of work, but there appears to be a relationship between RMB and resultant management.

One of the most significant criticisms of using RMB for management of SRM is concern regarding accuracy of the biopsy result. Some use the unreliable surrogate of growth rates to substitute for surgical pathology in confirming biopsy results. This has led to reluctance in adopting RMB as a standard. Dechet et al. (1999) conducted one of the first and largest studies to have surgical pathology confirmation of RMB. In this study, renal masses were biopsied twice with an 18-guage needle after surgical removal. These samples were processed with hematoxylin and eosin stains, compared to final pathology on the surgical specimen, and evaluated blindly by two independent uropathologists. Accuracy for the pathologists was 76% and 80%, with non-diagnostic rates of 11% and 17%, sensitivity of 77% and 84% specificity 60% and 73%, positive predictive value 94% and 96%, and negative predictive value 69% and 73%. In this ideal setting in which biopsies were performed *ex vivo* and then compared to the gold standard of surgical pathology, accuracy rates were lower than previously reported. This suggests that studies in which biopsy results are not confirmed by surgical pathology might lead to an overestimation of the accuracy of RMB.

In a more recent prospective study, Schmidbauer et al. compared pre-operative percutaneous fine-needle and core biopsy with surgical pathology in 78 patients and report more promising diagnostic ability (Schmidbauer et al., 2008). They reported sensitivity for the detection of renal cell carcinoma of greater than 90%, specificity of 100%, positive predictive value of 100%, and a negative predictive value of 70%. They were also able to correctly identify Fuhrman grade in 76%, and correct subtype identification in 91%. In contrast, a review of 405 preoperative biopsies from 378 patients from the MD Anderson Cancer Center from 1991 to 2007 showed concordant Fuhrman grades in preoperative biopsies and nephrectomy specimens in only 38.3% of patients (Abel et al., 2012). These data demonstrate the wide variability in accuracy rates of Fuhrman grade from RMB and suggest that determination of grade may not as useful in managing SRM until accuracy rates uniformly improve.

Improvements in accuracy rates in multiple studies are difficult to evaluate due to methodological differences in studies, but appear to be improving (Chawla et al., 2006; Kunkle et al., 2007). In the large meta-analysis by Lane et al. accuracy rates prior to 2001 were reported as 88.9% and 96% after 2001. They attribute this improvement to advances in molecular diagnostics.

## Advancements in renal mass biopsy

Some of the most significant limitations in relying on biopsy are the heterogeneous nature of SRM and the difficulty distinguishing masses based on histology alone. Recent identification of new histologic and molecular markers will undoubtedly change the sensitivity and specificity of subtyping these lesions and aid in deciding which lesions are safe to actively monitor and which need aggressive early management. Further, characterizing lesions based on molecular markers, chromosomal changes and gene expression profiling using PCR, fluorescent *in situ* hybridization (FISH), and microarray analysis will provide information which may help predict malignancy potential as well as sensitivity to immunotherapy and possibly chemotherapy (Table 1) (Martignoni et al., 2001; Young et al., 2001; Higgins et al., 2003; Takahashi et al., 2001; Yao et al., 2005; Zhou et al., 2005; Leppert et al., 2007; Varona et al., 2010; Koul et al., 2011; Lee et al., 2011; Samplaski et al., 2011). Molecular subtyping has already been shown to accurately predict malignancy potential and tumor sensitivities to direct chemotherapy regimens in colorectal and breast cancer, changing the way patients are managed. Similarly, serum carbonic anhydrase IX level, and expression levels of CD147, HIF1-alpha and VEGF have been associated with increased renal tumor progression and recurrence rates and may be helpful as predictors of poorer outcomes (Lam et al., 2005; Klatte et al., 2007; Sandlund et al., 2007; Li et al., 2008; Liang et al., 2009).

**Table 1:** **Molecular markers and genetic alterations of subtypes of renal cell carcinoma**.

**Renal cell carcinoma subtype**	**Molecular markers**	**Genetic alterations (Koul et al., 2011)**
Clear cell	(+): GST-α, Vimentin, ADFP, CA-IX, EMA, LMWCK, CD10, Caveolin-1, MOC-31, CD26	−3p25, +5q22, −6q, −8p12, −9p21, −9q22, −10q, −14q
	(−): K19, AMACR, Keratin 7, CK20, CK7, HMWCK, Ron, Parvalbumin	
Papillary	(+): AMACR, CA-II Keratin 7, CD 10, CD15, LMWCK	+3q, +8, −9p21, +12, −14q, +16, +17q21, +20
	(−): GST-α, CA-IX, Ron, Parvalbumin	
Chromophobe	(+): CA-II, Parvalbumin, CD74, Galactin-3, Cytokeratin 7, Caveolin-1, MOC-31, CK7, E-cadherin, CD10	−5q22, −8p, −9p23, −18q22
	(−): AMACR, K19, Vimentin, ADFP, HMWCK, Ron, CD26	
Oncocytoma	(+): CA-II, Parvalbumin, Ron, Galectin-3, CD 10, LMWCK, E-cadherin, Caveolin-1, CD26	−1p, −8p, −11q13, 14q, −19q, −21q, −X/Y,
	(−): GST-α, AMACR, K19, Vimentin, CD 74, HMWCK	der(13)t(13;16)(p11;p11)

One of the most difficult areas in the management of SRM is being able to histologically distinguish between benign oncocytomas and variants of chromophobe renal cell carcinoma on RMB. Oncocytomas are usually considered benign lesions while chromophobe tumors have a more malignant potential (Amin et al., 1997; Perez-Ordonez et al., 1997; Cheville et al., 2003). Although these two types of tumors are difficult to distinguish by cellular morphology, they can be distinguished using multiple defining histologic and molecular characteristics (Young et al., 2001; Kuroda et al., 2004; Adley et al., 2006; Garcia and Li, 2006; Huang et al., 2009; Samplaski et al., 2011), with published accuracy rates as high as 94% for distinguishing between renal cell carcinoma and oncocytoma when combining histology with FISH (Barocas et al., 2007).

Additional markers such as Caveolin-1, which was shown to be positive in 87% of chromophobe RCCs (20 of 23) and 0% of oncocytomas (0 of 8) and MOC-31, which was positive in 96% (22 of 23) of chromophobe RCCs and only 25% (2 of 8) of oncocytomas may also provide clinically important data (Lee et al., 2011). Moreover, different permutations of Cyclin D1 expression levels and rearrangement of the CCND1 locus can help differentiate oncocytomas and chromophobe renal cell (Sukov et al., 2009). Further advancements in determining unique cellular and molecular characteristics will help identify tumor subtypes, although the number of assays run on a single sample may be limited by the amount of tissue available from core biopsies.

Finally, it should be noted that improved accuracy rates are not only due to more molecular markers, but also likely due to experienced uropathologists familiar with the renal cell tumor histology. The interpretation of the biopsy results will be very dependent on the expertise of the pathologists. The role of renal biopsy is not proven amongst a general population of pathologists and therefore the interpretation should be limited at this point to pathologists with this expertise.

## Predicting tumor behavior

Currently, RMB is generally indicated for those with known extrarenal malignancy, suspected lymphoma, prior to and after ablation of a renal mass and to rule out an infectious etiology of a renal mass (Sahni and Silverman, 2009). Histological subtype and to a more varying degree, Fuhrman Grade can be determined by biopsy in most cases (Lechevallier et al., 2000; Neuzillet et al., 2004; Lebret et al., 2007; Shannon et al., 2008; Blumenfeld et al., 2010). However, even within these subtypes and grades, significant variation can occur in terms of aggressiveness, recurrence and susceptibility to certain treatments. Researchers are now looking towards genetic and protein profiling to not only help more accurately identify subtypes of tumors, but also aid in predicting outcomes, potential response to targeted systemic therapy, metastatic behavior and susceptibilities (Oda et al., 1995; Wu et al., 1996; Young et al., 2001). Barocas et al. (2006) reported results from 60 core biopsies taken after nephrectomy and found that histology alone had an accuracy rate of 83.3%, but in combination by PCR based gene expression techniques the accuracy rate improved to 95%.

Takahashi et al. (2001) identified expression of 40 genes associated with worse outcomes in clear cell renal carcinoma. Using this molecular profile, they were able to more accurately predict the course of disease (96% of cases) when compared to staging alone. In a recent study looking at cytogenetic profiles of patients with clear cell renal carcinoma, Klatte et al. identified that loss of certain chromosomes were tightly associated with better or worse outcomes.

Future work will continue to improve our understanding of which malignant tumors are likely to behave poorly, further aiding our ability to risk stratify patients with SRM. The Cancer Genome Atlas (TCGA) is an open resource that provides data on gene and miRNA expression, DNA methylation and copy number in several tumor types, including clear cell and papillary cell carcinoma. As the TCGA grows, it will become a valuable resource in understanding many aspects of renal cell carcinoma including pathways affected in these tumors, which will ultimately drive drug discoveries specifically targeting these pathways. This is especially important in renal cell carcinomas, as these tumors are notoriously poor responders to traditional chemotherapy. This database will certainly serve the community in a collaborative effort to further expand our understanding of how molecular biology can serve as a platform for providing better, patient-specific care. As the dream of personalized medicine becomes more of a reality, it is easy to envision how renal biopsy in larger, locally advanced lesions or those associated with metastatic disease will become integral in the management of the advanced forms of the disease (Abel et al., 2012). Targeted therapies are (and more will become) available for specific renal cell carcinoma subtypes and may prove to be more effective in the neoadjuvant setting rather than the adjuvant or salvage setting.

## Cost-effectiveness of renal mass biopsy

An important consideration in the discussion of RMB for SRM is the economic impact of various treatment approaches. After considering biopsy performance, the probability of tract seeding, possibility of growth of the SRM, treatment costs, patient outcomes, and quality of life, Pandharipande et al. (2010) compared RMB to surgery or imaging surveillance. Their Markov model clearly favored RMB in terms of cost-effectiveness.

In a separate analysis using similar methods, Heilbrun et al. estimated that for a hypothetical healthy 60 year-old man with a SRM <2 cm, RMB was more cost-effective than immediate treatment for quality adjusted-life years gained (Heilbrun et al., 2012). Both of these studies argue for additional consideration of RMB when faced with a SRM.

## Diagnostic value of imaging in renal masses

The alternative to utilizing a pre-treatment RMB is to depend solely on imaging information. Although most enhancing renal masses are malignant, there are no definitive characteristics of a renal mass on CT or MRI that can conclusively distinguish between malignant tumors from benign growths (Choudhary et al., 2009; Rosenkrantz et al., 2010). Moreover, RMB provides superior diagnostic accuracy when compared head-to-head with imaging. Dechet et al. conducted a study where two radiologists reviewed CT scans from 100 patients with a solid renal mass and these results were compared to those of pathologists reviewing core samples. Final diagnoses were determined by surgical pathology. Utilizing CT imaging alone, the accuracy rates for each radiologist were 60 and 66%, non-diagnostic rates were 31 and 23%, sensitivities were 70 and 77% and specificities were 20 and 20%, respectively. Pathologists reviewing core biopsy rates were superior in all categories with accuracy rates of 77 and 72%, non-diagnostic rates of 20–21%, sensitivities of 81 and 83% and specificities of 60 and 33% (Dechet et al., 2003). With improvements in RMB as outlined above, diagnostic accuracy may favor pathologist review even further.

Other work has evaluated the accuracy of imaging for the diagnosis of SRM with mixed results. A meta-analysis consisting of 2770 patients undergoing either partial or radical nephrectomy for SRM demonstrated a relationship between renal mass size and malignancy. As renal mass size increased, the probability of being malignant also increased with a 17 % increase in the odds of malignancy with each 1 cm in size (Frank et al., 2003). In contrast, a retrospective review of 543 patients who underwent surgical excision compared pre-operative imaging to final pathology. They found a negative malignancy rate of 14.7% and mass size did not predict final pathology with 83% of benign masses considered suspicious for malignancy based upon imaging (Remzi et al., 2007).

When examining fine needle and core biopsies of patients presenting for percutaneous ablation, Heilbrun et al. (2007) found imaging to have a positive predictive value of 95% for malignancy but a non-diagnostic rate of 11.8%. However, a similar study reported benign core biopsy pathology in 37% of patients with suspected malignant SRM referred for percutaneous ablation but their benign results may be falsely inflated as non-diagnostic biopsies that then later showed no growth on repeat imaging were categorized as benign (Tuncali et al., 2004). Unfortunately or primary neither study has surgical pathology for comparison to confirm the malignant diagnosis.

Considering the limitations of imaging alone to conclusively determine malignancy, it is hard to argue that RMB would not offer additional information helpful in improving diagnostic accuracy and aid in the decision process of who might benefit more from surgery.

## Complications of renal mass biopsy

RMB is not without risks and these should be considered when deciding whether to pursue this diagnostic procedure. However, the risks of RMB are low; the most common complications of RMB include bleeding, arteriovenous fistula formation and tumor seeding. Reported rates of minor complications from renal biopsy are less than 5%, major complications are less than 1% (Lane et al., 2008), and mortality rates are less than 0.1% (Kark et al., 1958; Slotkin and Madsen, 1962; Muth, 1965).

The most common complication encountered is bleeding, which is usually subclinical and detected on CT scan during follow up with self-limiting treatment. In one series, bleeding rates of 91% were reported, however, major bleeding that required transfusion or hospital observation occurred in only 1.5% of cases (Tang et al., 2002). Although it is believed that larger-needle biopsies (18 gauge or less) are associated with higher risk of bleeding complications than with smaller-needle biopsies (20 gauge or more), published studies refute this idea showing that there is no significant difference in bleeding complications based on needle size (Gazelle et al., 1992; Wood et al., 1999). Retrospective nonrandomized studies showing a difference in complication rates with relation to needle size are thought to be a result of biopsy technique rather than needle size (Manno et al., 2004). It should be noted when comparing needle gauge size and diagnostic yield, there was no difference between 18 and 20 needle gauge size (Beland et al., 2007).

Arteriovenous fistula formation is a complication observed in 1.5–16% of cases (Dorffner et al., 1998). However, a majority of these are self-limited and clinically insignificant. Approximately 80% of arteriovenous fistulas will resolve on their own in a period of 3.5–20 months without any intervention (Matsell et al., 1992; Parrish, 1992; Tzortzis et al., 1998). The remaining may have clinical symptoms such as hematuria, hypertension or alteration in kidney function and are usually managed with angioembolization (Kopecna et al., 2005).

Tumor seeding is a serious concern for any biopsy procedure. The risk associated with tumor seeding along the needle track in RMB is rare, with reports of less than 0.01% (Smith, 1991; Volpe et al., 2007). Transitional cell carcinomas may have a higher risk for seeding, but this risk is still thought to be low (Wehle and Grabstald, 1986; Shenoy et al., 1991; Keeley et al., 1997; Herts, 2000). There are additional reports using modern biopsy techniques that have no cases of tumor seeding even in the cases where transitional cell carcinoma was biopsied (Lechevallier et al., 2000; Caoili et al., 2002; Neuzillet et al., 2004; Vasudevan et al., 2006).

Another often discussed potential unintended consequence of RMB is that if surgical management is required, it may make doing so more complicated. However, increasing evidence suggests that previous biopsy does not result in increased surgical complications or negatively impact outcomes and should not be used as a reason for avoiding RMB (Wood et al., 1999; Lechevallier et al., 2000; Neuzillet et al., 2004).

## Conclusion

The role of RMB in the setting of SRM has been expanding, driven by the knowledge that approximately 20–50% of SRM removed by surgical excision have benign (or relatively indolent) pathology (Frank et al., 2003; Nguyen et al., 2006). Additionally, low complications rates of RMB encourage its wider adoption. Further, as RMB becomes a more routine part of management of SRM, physicians will become increasingly skilled at this procedure, likely decreasing failed biopsy rates. Ongoing research continues to show promise in the development of molecular, cytologic and histologic markers to further characterize SRM and determine immunotherapy or targeted systemic therapy suceptibility, predict tumor behavior and outcomes, and discover new pathways in renal tumor biology.

The selective utilization of RMB for diagnosis in renal masses is a relatively uncommon approach when compared to management of other neoplasms. In most other solid tumors, obtaining a biopsy is one of the first steps in evaluating a patient and in making an informed treatment decision. Clinicians can increasingly risk-stratify patients based upon RMB results, leading to important decisions such as whether to excise the tumor, likely safety of AS, and potentially which type of systemic treatment regimen to employ. With this ever-increasing data on the usefulness of RMB, it may be time to increase utilization as part of routine practice in the management of the SRM.

### Conflict of interest statement

The authors declare that the research was conducted in the absence of any commercial or financial relationships that could be construed as a potential conflict of interest.
